# An exploratory study of the experiences and challenges faced by advanced life support paramedics in the milieu of neonatal transfers

**DOI:** 10.4102/hsag.v26i0.1562

**Published:** 2021-10-28

**Authors:** Raisuyah Bhagwan, Pradeep Ashokcoomar

**Affiliations:** 1 Department of Community Health Studies, Faculty of Health Sciences, Durban University of Technology, Durban, South Africa; 2 Department of Emergency Medical Care, KwaZulu-Natal Department of Health’s Emergency Medical Services College, Durban, South Africa

**Keywords:** neonates, transfers, education, ALS paramedics, KwaZulu-Natal

## Abstract

**Background:**

The safe transfer of critically ill neonates is important for their survival. This calls for greater preparedness on the part of paramedics to effect these transfers safely.

**Aim:**

To understand the experiences and the challenges faced by advanced life support (ALS) paramedics during neonatal transfers.

**Setting:**

The study setting consisted of advanced life support paramedics from urban and rural areas in KwaZulu-Natal. It comprised of a network of district hospitals as well air and ground transfer facilities, both public and private.

**Method:**

Using a qualitative research approach, the study sought the views of ALS paramedics who were involved in neonatal transfers in KwaZulu-Natal. A purposive sample of *n* = 8 ALS paramedics was selected. Data were collected using in-depth semi-structured interviews. The data were analysed through the process of thematic analysis.

**Results:**

The study found that paramedics faced multiple complex challenges related to neonatal transfers. Poor pre-transfer preparation of the neonate, equipment related challenges, lack of clinical support available during transfers and pressure to effect inappropriate transfers were some of the challenges they faced. These challenges coupled with insufficient education and the lack of sub-speciality programmes to capacitate, rendered them unprepared to deal with neonatal transfers effectively.

**Conclusion:**

Emergency medicine needs to provide greater attention towards preparing all stakeholders for successful neonatal transfers.

**Contribution:**

The findings provide recommendations for a programme that will limit risks involved with, and support the inter-healthcare facility transfer of critically ill neonates in South Africa.

## Background

A neonate is a term used in the international literature to refer to infants, specifically from birth through the first 28 days of life. Neonates can be further categorised into newborns (at the time of birth), early neonates (from birth to 7 days of life) and late neonates (from 7 to 28 days of life) (Smith et al. 2009). Critically ill neonates are those who have life-threatening illnesses associated with single or multiple organ system failure (Whyte & Jefferies 2015). The inter-facility transport of critically ill neonates is an integral component of emergency medical services in South Africa. The treatment of sick neonates in more specialised neonatal intensive care units (NICUs) has been linked to decreased morbidity and mortality (Pai et al. 2020). Transfers become crucial when neonates are born in hospitals that are ill-equipped or ill-prepared to deliver specialised neonatal intensive care (Wahab et al. 2019).

Research in South Africa has found that the likelihood of an adverse event occurring during the transportation of neonates is at least 75% (Pan 2017; Sabzehei et al. 2016). Apart from transportation, the change of environment from an ambulance to a specialised NICU adds additional stress to the neonate, commonly resulting in either minor or life-threatening events (Chakkarapani et al. 2020). Physiologically related adverse events are directly linked to the clinical deterioration of the neonate, with physiological changes being a real possibility during both ground and air transportation (Sethi & Subramanian 2014). The common physiological changes that result in the clinical deterioration of neonates include hypothermia, hypotension, hypoglycaemia, cardiac dysrhythmia, respiratory deterioration and decreased levels of consciousness (Pai et al. 2020). Goldsmit et al. (2012) documented that 49 (31%) of the 91 neonates who experienced physiological changes during transport, required immediate cardiorespiratory support. Thermal instability was the most common adverse reaction, with 73 (46%) of the neonates deteriorating as a result (Goldsmit et al. 2012). In South Africa, although there is limited literature on inter-healthcare facility transfers of critically ill neonates, evidence that exists reflects cause for much concern. Mgcini (2011) who conducted a cross-sectional, descriptive study of 104 transfers by both private and public ambulances in Gauteng province from October to December 2007 found that many neonates arrived in a poor clinical condition following transfer to a referral hospital. This resulted in a relatively high mortality rate (7%) occurring within 48 h after the transfer with the most significant predictors of mortality being bradycardia, hypoxia, hypotension and hypothermia. Mgcini (2011) listed the common adverse clinical events noted on arrival at the receiving hospital as hypotonia (32%), hypoxia (22%), hypothermia (21%) and acidosis (40%). Similar findings were made by Ashokcoomar and Naidoo (2016), whose study found that of the 29 (24.2%) critically ill neonates transferred, 10 (8.3%) had physiologically related adverse events during transfers, including one (0.8) death. The remaining nine incidents were all potentially life-threatening and included conditions such as respiratory and cardiac deterioration, desaturation, the development of hypothermia and cardiorespiratory arrest. The causes of the neonatal death included inappropriate pre-transport preparation, lack of pre-transfer stabilisation and a lack of available ALS paramedics (Ashokcoomar & Naidoo 2016). These findings are similar to the international findings from developing countries, such as India (Deepak, Adhisivam & Vishnu 2015) and Jamaica (Henry & Trotman 2017), where physiological changes, such as hypothermia and hypoglycaemia were commonly reported during transport. These studies concluded that the resulting issues could have been prevented had neonates been stabilised prior to departure.

Critical care and monitoring of a critically ill neonate during the transfer process is provided by an advanced life support (ALS) paramedic. These paramedics are registered as either Critical Care Assistants (CCA), National Diploma in Emergency Medical Care (NDEMC) or Emergency Care Practitioners (ECP). These ALS paramedics play a vital role in the emergency medical care (EMC) environment as they are the pre-hospital specialists and currently responsible for intensive care during the neonatal transfer (Health Professions Council of South Africa [HPCSA] 2014). Their scope of practice enables them to provide a wide range of intensive care interventions including, but not limited to, advanced airway management and ventilation, cannulation (intravenous, intraosseous and umbilical), pharmacological administration, emergency cardiovascular care and advanced cardiac arrest management.

The aim of this research was to analyse the current inter-healthcare facility transfer of critically ill neonates in KwaZulu-Natal and to investigate the state-of-the-art practice that guides the transfer of critically ill neonates. Moreover, this study explored the views of ALS paramedics on all aspects that need to be operationalised in a programme that will guide the inter-healthcare facility transfer of critically ill neonates in South Africa.

## Methodology

### Study design and setting

A qualitative, explorative and descriptive design was used. The study area involved 11 health districts in the province of KwaZulu-Natal. This was the most accessible province in terms of the study and was deemed appropriate as it comprised both urban and rural areas, a network of district hospitals, as well as air and ground transfer facilities, both public and private. To guide the report of findings, the consolidated criteria for reporting qualitative research (COREQ) standards were followed (Buus & Perron 2020).

### Population and sampling

The target population for the study included all ALS paramedics who were working in the province of KwaZulu-Natal. Non-probability sampling methods, more specifically purposive sampling was used as a sampling strategy to purposefully select the paramedics. Non-probability sampling methods, specifically a convenience sampling approach was the strategy used. Convenience sampling allows for the purposeful selection of participants who can provide rich information related to the objectives of a study (Etikan, Musa & Alkassim 2016).

### Sample: Advanced life support paramedics

The researcher forwarded a detailed letter (via email, post or fax) containing information regarding the study and its requirements to operational ALS paramedics who were directly involved in neonatal transfers, inviting them to participate in the interviews. The ALS paramedics who undertook transfers over a period of 1 month, from 01 to 31 December 2015 were recruited to participate in the study. Thirty-one paramedics were found to have been involved in these transfers and they were all selected as part of a preliminary analysis that focussed on providing an overview of the transfer context and its dynamics. Eight paramedics were then purposefully selected, so that there were some representations of those involved in neonatal transfers in urban (6) and rural (1) areas, and there was representation from those involved in public (7) and private (1) transfers with use of either ground (7) or aeromedical ambulances (1). Six participants were CCA and two were ECP who had either a Bachelor of Technology in Emergency Medical Care (EMC) or Professional Bachelor’s degree in EMC. Moreover, the paramedics selected were those who were involved in more than one transfer for that month. The inclusion criteria included ALS paramedics who had completed the preliminary analysis and were willing to participate in an individual interview. This included operational ALS paramedics working in the public emergency medical services (EMS) and one major private EMS (Netcare). ALS actively involved in transporting critically ill neonates. ALS from which gatekeeper approval was granted by 8 of the 9 provinces (KwaZulu-Natal, Eastern Cape, Free State, Gauteng, Limpopo, Mpumalanga, Northwest and Western Cape).

The exclusion criteria excluded ALS paramedics who participated in the situational analysis but did not accept the invitation to an individual interview, non-operational ALS paramedics, operational ALS not actively involved in transporting critically ill neonates. The second major public EMS did not consent to participate. Northern Cape province did not grant gatekeeper approval because of limited ALS in the province.

### Ethical considerations

Ethical clearance for this study was obtained from the Institutional Research Ethics Committee (IREC) of the Durban University of Technology (DUT). The study was allocated ethical clearance number: IREC 093/15. Permission was thereafter obtained from all Provincial Departments of Health via the National Health Research Database (Reference number: FS 2015RP12685) and the private sector EMS head office.

All participants were informed via email correspondence about the study and then telephonically, before data collection they were told that their participation was voluntary and that they could withdraw at any given time. Written consent was obtained from all participants before the interviews commenced. Moreover, they were all assured of their anonymity in the process and that their identifying details would remain confidential. They were also informed that there were no financial benefits through participation but that the findings would be disseminated through publications. Compensation was not provided for the participants in this study.

### Researcher background

The researcher was a trained ALS paramedic who had extensive experience in the neo-natal inter-hospital transfer system in KwaZulu-Natal. As a former Principal of the KZN College of Emergency Care, he was a well-established researcher in the Department of Health and was guided by core values of integrity, respect and accountability. He has had previous experience in terms of research regarding neonatal transfers and has successfully accomplished a Master’s Degree by undertaking a quantitative survey study, which explored the dynamics associated with this type of transfer. There was no language barrier between the researcher and the participants. Moreover, there was no personal relationship between the researcher and the participants.

### Data collection process

Following study enrolment, the researcher made logistical arrangements (i.e. setting times and venues to meet the participants) within the 11 targeted districts to ensure participant availability and to provide them with directions to the venue. The researcher also contacted the participants telephonically to provide more specific information about the study and to answer any questions regarding the participation. The interviews were conducted in natural settings that were comfortable for the participants and conducive for recording. These included boardrooms and offices at Department of Health premises where the participants worked.

Data was collected from the sample using in-depth semi-structured face to face interviews. This enabled the collection of rich data from the ALS paramedics regarding their experiences and the challenges confronting them during the transfer process. An interview guide with open-ended questions, using probes as necessary, was used as the data collection instrument.

The questions asked were as follows:

1. What are the multidimensional issues that challenge the safe inter-healthcare facility transfer of critically ill neonates?
2. What are the state-of-the-art practices that guide the inter-healthcare facility transfer of critically ill neonates?
3. What are the views of ALS paramedics, family members, neonatologists and EMC lecturers on what needs to be operationalised in a programme for the inter-healthcare facility transfer of critically ill neonates in South Africa?
4. What are the components of a multidimensional programme that will enable a successful transfer?

The interview schedule was designed in accordance with the objectives of the study, which was to analyse the current inter-healthcare facility transfer of critically ill neonates in KwaZulu-Natal. The instrument was pilot tested and refined with a group of five ALS paramedics who were not involved in the current study. It was modified based on the recommendations made. In all instances, the researcher collected the data, made field notes, kept a journal and used an audio tape to record information collected. All interviews were audio recorded using a zoom recorder and lasted between 60 and 90 min. Audio recordings were transcribed verbatim. Interviews were stopped only once data saturation was achieved.

### Data analysis

Thematic analysis was used to analyse the data using the steps outlined by Braun, Clark and Hayfield (2019). The research was involved in the entire coding process. This approach allowed the researcher to make sense of the collective meanings and experiences of the paramedics and to organise and reduce it into themes and sub-themes. A preliminary coding scheme was generated which served as a template for the data analysis (Tutty, Rothery & Grinnell 1997). Similar themes and recurring patterns in the data were then linked together and the contrasts and differences identified (Liamputtong & Ezzy 2005).

### Trustworthiness

To enhance the trustworthiness of this study, the researcher made use of Guba’s model (1994). This model provides four criteria to ascertain rigour in qualitative studies, namely credibility, dependability, conformability and transferability. Strategies used were multiple interviews with ALS paramedics, member checking, using the triangulation process during data analysis and maintaining an audit trail and a process journal (Buetow 2019). This ensured trustworthiness of data and reflexivity of the researcher. In addition, an expert evaluation committee was also used to validate the findings made.

## Results

### Demographics information

The demographic profile of the participants is represented (Table 1).

**TABLE 1:** Demographic profile of the participants.

ALS paramedics sample size	Sector	Ambulance	Geographical location	Qualification
8 × Participants	7 × Public sector	7 × Ground ambulance	6 × Urban area	6 × CCA
	1 × Private sector	1 × Air ambulance (public)	1 × Rural area	2 × ECP
			1 × Aeromedical	

ALS, advanced life support; CCA, Critical Care Assistant; ECP, Emergency Care Practitioner (Bachelor of Technology in EMC or Professional Bachelor Degree in EMC).

### Themes

There were two broad themes that emerged in relation to the objectives of this research. The first theme focussed on the challenges associated with the transfer process and the second theme focussed on the need for specialised knowledge and skills. Five sub-themes were derived from theme one, namely pre-transfer challenges, preparation of the neonate, the transfer journey, the lack of clinical advice and pressure to effect inappropriate transfers. There were two sub-themes that emerged under theme two and these included inadequate educational preparation and a lack of subspeciality programmes.

#### Theme 1: Challenges associated with the transfer process

The participants reported several challenges experienced within the transfer processes. These will be described within the context of the following four sub-themes:

**Sub-theme: Pre-transfer challenges:**

‘Sometimes control gives us false information. When you go to hospital you realise that the doctor requested equipment, but they didn’t tell us. We now have to go back to get the equipment or make a plan.’ (P2)

‘As far as the diagnosis… I quickly do some research on what the pathology is, that I’m dealing with, quickly go over the infusions and stuff like that, so I have a better understanding. Neonatal transfers are hectic you know, so we need all the information to do all of this.’ (P6)

The participants identified two crucial issues that created challenges prior to the transfer of the critically ill neonates. The first reflected the lack of accurate information pertaining to the critically ill neonate. Faced with this, paramedics have to return to secure the requisite equipment which creates time delays or alternatively, ‘make a plan’ which may mean undertaking a transfer without the requisite equipment. Both scenarios highlight the current dynamics within the transfer system.

In addition to being plunged into a situation without accurate factual information, their ability to be prepared to deal with the presenting clinical emergency of the ill neonate was highlighted. This was evident in the voice of one paramedic who described such transfers as ‘hectic.’ This participant emphasised the need not only for accurate information, but as much information as possible so that they could be empowered to manage the transfer effectively. The fact that paramedics had to quickly read on the presenting pathology highlights the fact that they are sometimes unprepared for the clinical challenges associated with a specific transfer.

**Sub-theme: Preparation of the neonate:** The participants highlighted several issues that emerged during the pre-transfer preparation of the neonate that included the following:

‘Often critically ill neonates are not packaged properly or not packaged at all. We need to package patients appropriately before leaving the hospital. Sometimes the hospital staff are not familiar with the procedures we have to undertake.’ (P2)

‘There is a lack of skills and knowledge even by doctors that is really hampering or making life difficult for us, especially in referring hospitals, at the district and rural hospitals. They do not intubate and ventilate the patient because they wait for us to come to treat the babies. It’s either that they are not confident with their skills or they don’t have equipment or don’t understand their equipment.’ (P2)

‘Critical babies don’t do very well in flight, therefore they must be stable before the flight. Once we take off, we cannot just pull over and stabilise the patient. The patient must be fully stabilised for flight.’ (P5)

One of the participants said that critically ill neonates are ‘often’, not packaged properly in preparation for the transfer process. This highlights the possibility for time lost when this has to be performed by the paramedics and further shows that other medical personnel are unaware of what needs to be performed in preparation for the transfer. This notion was reiterated by another participant who reported that doctors themselves do not intubate or ventilate the neonate and prefer to hand over this intervention to the paramedic to perform.

Another participant highlighted the danger of inadequate stabilisation of the neonate particularly prior to an air transfer. He cautioned that critically ill neonates do not do well during such transfers and said that when clinical emergencies occur during a flight, it becomes challenging to try to stabilise the neonate.

**Sub-theme: The transfer journey to the hospital:** The transfer journey itself was found to present several challenges for the paramedics. They reported as follows:

‘There’s no one assisting me, if something does go wrong then I’m stuck with the patient alone. When we do the transfer with crews, the transfer works well and is not so much of a hassle.’ (P3)

‘Equipment moves all over, We try our best to secure the equipment but our ambulances are not properly designed…equipment failure during transport is our biggest issue right now.’ (P7)

‘We do have problems sometimes when you get to hospital the doctors are not aware the patient is coming through. The doctor and nurse only start preparing the bed and the ventilator when we [are] there, rushing and doing things like that, the doctor was not informed that the patient is coming through. So that’s delay, you have to wait half an hour. We run out of oxygen sometimes while waiting to hand over.’ (P3)

The heightened fear and anxiety of managing a critically ill neonate alone during a transfer was evident in one of the participant’s voices. The participant pointed out the danger associated with endeavouring to care for a critically ill neonate in the event of an emergency, stating, ‘I was literally “stuck”’. The participant further affirmed that those transfers that are undertaken with other paramedics are successful, as opposed to being confronted with a clinical emergency alone where difficulties arise in trying to keep the fragile neonate stable. These risks were further exemplified in the second excerpt where a paramedic expressed the difficulties that arise when the equipment moves around during the transfer. He also brought to the fore the problems associated not only with using an ambulance that is not designed for the transportation of ill neonates but also the additional burden of encountering malfunctioning equipment, within the context of trying to ensure the survival of the ill neonate.

The third excerpt highlighted immense stress experienced by paramedics who after completing the transfer are faced by the un-preparedness of the hospital to receive the baby. One participant said that the doctors and nurses only begin to prepare the bed and ventilator when they arrive, thereby losing crucial time. This is linked to the earlier findings that the communication centre does not relay information that is both accurate and timely within the context of the transfer. All of these challenges may jeopardise the neonate’s chances for survival.

**Sub-theme: Lack of clinical advice:** One participant also expressed the need to secure clinical advice during the transfer journey as follows:

‘There’s no clinical advice. If I need clinical advice… if the baby deteriorates, then I would be stuck. I wouldn’t know what to do because we have no cell phones, no doctors contact numbers. Our only communication is through the control room but that is an issue on its own. I have never got[ten] clinical advice in all my years as a paramedic.’ (P3)

The aforementioned excerpt highlights the fear and anxiety ALS paramedics face when confronted by the deterioration of an ill neonate. It brings to the fore the lack of educational preparedness to deal with the clinical emergencies common to critically ill neonates. Moreover, it exemplifies their ‘isolation’, once the transfer begins as they have little opportunity to reach out to an expert for emergency assistance. It affirms the need for clinical support to be available to paramedics, who are faced with having to transfer a critically ill neonate.

**Sub-theme: Pressure to effect inappropriate transfers:** Paramedics also experienced pressure from several stakeholders to effect transfers, even when they did not appear to be appropriate:

‘You know, at times I feel much intimidated because I deal with professionals, handing over and taking over the babies from me.’ (P5)

‘On the aeromedical unit we are always pressured and sometime forced to do transfers, even if the neonate is unstable for transfer. This also happens on the ground unit but not as bad when working aeromedical. As much as we advise … that the neonate is unstable for transfer, they insist that we continue. We don’t have the power to disagree because the air ambulance is an expensive unit. Complaints escalate to high levels and we will get in trouble for refusing the case.’ (P6)

‘No one speaks for us. We get pressured from the aeromedical coordinator and EMS managers who make poor decisions based on what information is given to them telephonically, yet they do not listen to us even though we are with the patient and describing what we are seeing. We stress that this is an unstable patient for transfer but we are forced to do the case.’ (P6)

The paramedics expressed that they were ‘voiceless’ within the context of the transfer process. They reported that they were often pressured by various stakeholders into transferring unstable neonates with life-threatening conditions. The paramedics who felt coerced to undertake such transfers experienced feelings of helplessness especially when they were aware of the risks associated with the transfer.

#### Theme 2: Specialised knowledge and skill

The second theme that emerged from the data is related to specialised knowledge and skill. The sub-themes that emerged are related to inadequate educational preparedness of ALS paramedics and the lack of sub-speciality programmes to keep them abreast of neonatal transfer demands.

**Sub-theme: Inadequate educational preparedness:** All participants expressed that they lacked adequate educational training for neonatal transfers:

‘Due to the lack of a comprehensive programme with the correct equipment, staff, education and training, we are simply not prepared to deal with these critical babies the way we correctly should.’ (P2)

‘Sometimes I cannot answer the doctor’s questions…sometimes I have no comment because we are not prepared enough.’ (P5)

‘Nervousness will always be there because you are dealing with a neonate that has a life-threatening condition, you don’t know what to expect next… [*we are*] insecure because we are doing what we can. Transferring sick babies is my greatest fear because we are not prepared.’ (P1)

**Sub-theme: The lack of subspeciality programmes:** The paramedics highlighted that the lack of a subspeciality programme compromised their ability to gain knowledge and experience related to neonatal transfers:

‘We are transferring critical patients from neonatal intensive care units, it’s a speciality on its own. We are taking over and handing over from specialists, neonatologists, NICU nurses. We are the weakest link, to strengthen the link we must have subspeciality programmes.’ (P2)

‘I believe that all paramedics should be given an opportunity to choose a subspeciality within their scope of practice, this is common practice elsewhere but not the case in EMS.’ (P4)

‘The staff must have experience in neonatal transfers and want to work on the unit, not forced to do the transfers. Certain paramedics prefer trauma patients [*or*] medical patients… so working on the neonatal transfer unit should be the paramedic’s choice because he wants to do work there.’ (P7)

These statements highlighted the fear and anxiety that paramedics experienced during transfers because of their perceived lack of preparedness and training to deal with neonatal emergencies.

## Discussion

This study highlighted several findings that were of concern. Poor pre-transfer preparation of the neonate, equipment related challenges, lack of clinical support available during transfers and pressure to effect inappropriate transfers were some of the challenges they faced. These challenges coupled with insufficient education and the lack of sub-speciality programmes to capacitate, rendered them unprepared to deal with neonatal transfers effectively. The findings highlighted two broad themes, that is, challenges associated with the transfer process and specialised knowledge and skills.

Theme one presented the following sub-themes: pre-transfer challenges, preparation of the neonate, the transfer journey to the hospital, lack of clinical advice and pressure to effect inappropriate transfers. The first sub theme, pre-transfer challenges, reflected the lack of accurate information pertaining to the critically ill neonate and undertaking transfers without requisite equipment. Paramedics have a sense of helplessness because of these difficulties and the reality of the threat to the survival of the already fragile neonate. In this vein, Whyte and Jefferies (2015) asserted the importance of the Emergency Medical Care Centre receiving as much information as possible about the neonate and the transfer requirements, so that this information can be accurately relayed to the team leader. Similarly, Stroud, Trautman and Meyer (2013) argued that the staff managing the Emergency Medical Care Centre should be well trained in neonatal transfers, and to receive calls and provide accurate information regarding transfer requirements so that there is proper preparedness for the transfer. In addition, the dispatch details given by the communication centre to the transfer team leader should be audible, and clearly understood, to avoid misunderstandings. Communications should include information about the neonate’s condition, the treatment already undertaken, equipment required and any other information vital for the transfer (Boxwell 2010). This inevitably creates a level of stress and anxiety for those paramedics who have to quickly read in anticipation of difficulties that may arise during the transfer.

The findings with regard to the preparation of the neonate, under sub-theme two highlighted the doctor’s lack of knowledge and expertise with regard to such procedures, particularly related to critically ill neonates. More importantly, however, it exemplifies the need for ALS paramedics to have the requisite training and sound experience in being able to undertake intubation and ventilation. Foglia et al. (2019) described neonatal tracheal intubation as a potentially dangerous procedure and argued the need to identify factors to improve neonatal intubation. The issues in the data highlight the need for ALS paramedics who are confronted with the transfer of a fragile neonate and have to deal with the unpreparedness of the hospital and other medical personnel associated with the transfer.

Messner (2011) supported this finding saying that pre-transfer preparation was a critical aspect of identifying the factors that may potentially arise and compromise the survival of neonate, before they are transferred out of the referring facility. This stabilisation was crucial to their ability to tolerate the transfer and to ensure a successful outcome (Carreras-Gonzalez & Brió-Sanagustin 2014). Hence, pre-transfer preparation of the neonate, is a vital component of the transfer which is crucial to ensuring the survival of the neonate during the transfer (Kumar et al. 2010).

The third sub-theme, that is, the transfer journey to the hospital, highlighted the fact that managing a critically ill neonate is a complex process, as the paramedic has to monitor the baby’s condition during the journey, in particular ventilation and oxygenation, thermal, cardiovascular and metabolic support and provide lifesaving interventions if necessary (Chakkarapani et al. 2020). This suggests the need for greater respect and support for the views of paramedics, when decisions are made within the transfer process.

The data also reflected in sub-theme four, that paramedics are left powerless within what appears to be a hierarchical work environment, which then compels them to follow instructions, from those in authority even when they believe a transfer should not be performed. Moreover, a lack of knowledge amongst these health care professionals, who may not always have sufficient information and expertise related to neonates, may be potentially dangerous when they issue instructions to undertake risky transfers. As evidenced, paramedics often receive instructions to issue transfer, regardless of the fact that they have not physically assessed the neonate. What was particularly concerning is that these paramedics often felt they had to implement the transfer regardless of them indicating that the neonate was unstable.

The fifth sub-theme namely, pressure to effect inappropriate transfers, reflected the pressure that paramedics received from several stakeholders to effect transfers. The literature also reflects that a ‘swoop and scoop’ is no longer considered appropriate, with speed being viewed as detrimental, in comparison to investing time in resuscitating and stabilising the neonate before the journey. Gilpin and Hancock (2016) supported this notion saying that pre-transfer stabilisation and preparation was perhaps more beneficial than rapid delivery to a healthcare facility. The objective of the transfer team should therefore be to achieve physiologically acceptable haemodynamic and metabolic parameters before transportation. Hence, transfers should not be performed hastily and panic transfers may result in morbidity or mortality. As Messner (2011) said, it is the hospital or clinic that provides a less risky environment for stabilising a critically ill neonate, than the challenging ride in the back of an ambulance or in a restricted space of a noisy unstable aircraft. Hence, everyone involved in the transfer process from beginning to end, including doctors, nurses, emergency care providers and support services should have a thorough understanding of the risks and processes involved, so that an informed decision is made, with a positive outcome for the neonate (Ratnavel 2013).

Theme two presented the following sub-themes: inadequate educational preparedness and the lack of subspeciality programmes. The sub-theme related to inadequate educational preparedness highlighted concerns around the inadequate educational training for neonatal transfers. This lack of unpreparedness rendered paramedics the ‘weakest link’, in a transfer chain where other professionals have more specialised knowledge and skills relevant to NICU. Moreover, they expressed the need for paramedics to secure relevant specialised experience related to transfers. One participant further suggested the need for paramedics to express their preference to work specifically with neonatal transfers. They added that some paramedics prefer trauma patients or medical patients and argued that those with a personal preference for this specialised type of transfer, be considered for this type of work.

The final sub-theme highlighted that the lack of a subspeciality programme compromised their ability to gain knowledge and experience related to neonatal transfers. In more developed countries, this appears to be implemented as neonatal transfer team members are selected on the basis of interest in the field and are capacitated with specialised education and training to ensure that they can provide an acceptable level of care that is beneficial to the outcomes of the neonate (King et al. 2007). In the absence of subspeciality programmes in South Africa (Ashokcoomar & Naidoo 2016), paramedics remain unprepared for neonatal emergency care.

The data also reflected that paramedics involved in neonatal transfers should be able to have a choice in terms of undertaking such work. A lack of educational preparedness coupled with a lack of expertise, derived from rigorous experience gained from being part of a neonatal transfer team, compromises their ability to be competent within the context of neonatal transfers. This suggests the need for adequate educational preparedness prior to paramedics becoming involved in this specialised area of practice.

What also emerged as important was the need for sub-speciality programmes so that paramedics could be capacitated on an ongoing basis. The intensive care unit (ICU) is a technologically sophisticated environment, which provides complex and detailed health care for various acute life-threatening conditions (De Beer, Brysiewicz & Bhengu 2011). Specialised knowledge and experience to function within this milieu is important to capacitate paramedics. Sub-speciality programmes are also important in terms of enabling further and continuous clinical preparedness for neonatal transfers.

Once ALS paramedics qualify, they register with the HPCSA and can immediately enter the field of EMS, with no internship programme that enables a level of preparedness for emergency transfers of critically ill neonates. Hence, whilst they may enter the workplace with a broad theoretical base and skills acquired through supervised experiential learning, the data suggest that this is insufficient to deal with the complexities of critically ill neonates. This places them at disadvantage, to those doctors or nurses in the NICU’s, in terms of both clinical decision-making and competence with regard to both transfer decisions and the ability to work effectively should the neonate deteriorate.

Mothers, who accompany neonates may also present with clinical emergencies during the transfer as they are often still patients themselves. Training under the supervision of a competent paramedic who is conversant with all these issues is crucial before those without clinical expertise should be allowed to effect neonatal transfers.

## Limitations

The interviews were covered only with the paramedics from KwaZulu-Natal. Whilst this is offset by the fact that qualitative inquiries seek information richness from small samples, a national inquiry would have shed greater light on issues across South Africa. Moreover, the sample consisted of mainly participants from the public sector and urban areas. Hence, the results cannot be generalised to the private sector and rural areas.

## Conclusion

The study revealed several challenges confronting paramedics faced with neonatal emergency transfers. These ranged from issues related to poor preparation and stabilisation of the neonate prior to the transfer and the lack of important information with regard to the neonate’s condition, which was crucial, so that paramedics could prepare themselves for dealing with the unique needs of the neonate. The study found out that the neonate was also not adequately stabilised prior to transfer and there were suggestions of a rushed transfer that could potentially become life threatening. The transfer journey itself appeared to have its own challenges.

Registration with the HPCSA demands a high level of professional competence to avoid negligence, as per statutory requirements. Given that the data suggest that EMC education may not be providing adequate preparedness for neonatal transfers, it is crucial that ongoing training in conjunction with a period of supervised practice with competent team members be initiated. Training should ideally cover stabilisation and resuscitation of the neonate, clinical skills that cover all emergency scenarios, use of specialised equipment, ambulance and aeromedical environment, safety guidelines and protocols, communication between relevant stakeholders, multi-disciplinary teamwork, debriefing, ethical issues and trauma support services for accompanying mothers. The latter should also enable paramedics to be well prepared for neonatal transfers.
